# 5 year follow up of a hydroxyapatite coated short stem femoral component for hip arthroplasty: a prospective multicentre study

**DOI:** 10.1038/s41598-023-44191-7

**Published:** 2023-10-11

**Authors:** Monil Karia, Kartik Logishetty, Hardeep Johal, Thomas C. Edwards, Justin P. Cobb

**Affiliations:** MSk Lab, Imperial College London, 2nd Floor, Sir Michael Uren Hub, 86 Wood Lane, London, W12 0BZ United Kingdom

**Keywords:** Medical research, Outcomes research

## Abstract

Short stem, uncemented femoral implants for hip arthroplasty are bone conserving achieving stability through initial metaphyseal press-fit and biological fixation. This study aimed to evaluate the survivorship, mid-term function and health related quality of life outcomes in patients who have undergone total hip arthroplasty (THA) with a fully hydroxyapatite coated straight short stem femoral component with up to 5 years follow-up. 668 patients were recruited to a multicentre study investigating the performance of the cementless Furlong Evolution^®^ stem for THA. 137 patients withdrew at various time points. The mean follow-up was 49 months. Clinical (Harris Hip Score (HHS), radiographic and patient-reported outcome measures—Oxford Hip Score (OHS) and EuroQol 5D (EQ-5D), were recorded pre-operatively and at 6 weeks, 6 months, 1 year, 3 year and 5 year follow ups. At 5-year follow-up, 12 patients underwent revision surgery, representing a cumulative revision rate of 1.8%. Median OHS, HHS and EQ5D scores improved significantly: OHS improved from a pre-operative median of 21 (IQR 14–26) to 47 (IQR 44–48) (p < 0.001). HHS improved from 52 (IQR 40–63) to 98 (IQR 92–100) (p < 0.001) and EQ5D improved from 70 (IQR 50–80) to 85 (IQR 75–95) (p < 0.001). This fully HA-coated straight short femoral stem implant demonstrated acceptable mid-term survivorship and delivered substantial improvements in function and quality of life after THA.

## Introduction

Total hip replacement (THR) is a highly effective procedure in relieving pain and restoring function for various hip pathologies^1^. Whilst cemented THR in elderly and low demand patients is particularly effective, failure rates are greater in the younger and more active patients^2,3^, most commonly occurring from aseptic loosening, endosteal bone lysis and occasionally from fatigue of the cement. As such, uncemented femoral stem implants are increasingly popular. They are used in almost 40% of all total hip arthroplasty in the UK and are the dominant choice in North America and Australia^4^. The global burden of hip arthroplasty is increasing, and bone-conserving solutions with longevity and excellent function are required particularly for younger and more active patients who are more likely to require revision surgery^5,6^.

Conventional length fully porous coated uncemented femoral stems have been associated with proximal femoral shielding and thigh pain after THA^5^. Subsequent revision can be technically challenging due to the biological distal fixation between the stem and host bone. To account for these shortcomings, shorter bone-conserving femoral stems (typically < 120 mm in length^6,7^ compared to a standard length of ~ 150 mm^8^ have been designed to preserve femoral bone stock and obtain a press-fit for metaphyseal loading. Although most uncemented stems will never need revision, in the event of revision, removal of a short stem may be theoretically less traumatic, with more native diaphyseal bone stock available for any subsequent implant. With many short stem implants now available, many have demonstrated survival^6^, clinical^9^ and kinematic function^10^ comparable to conventional length stems as well as reduced thigh pain^11^ although certain designs have shown unacceptably high revision rates.

Hydroxyapatite-ceramic (HAC) coating has become a well-established method of developing a biologic bond between the implant and patients bone in uncemented total hip replacements to improve osseointegration and primary stability^12–14^. The JRI Furlong^®^ HAC femoral component (JRI Orthopaedics Ltd, London, UK) is one such implant which has demonstrated good long-term survivorship^15–18^. It is manufactured from a titanium alloy (Ti-6Al-4 V), and the body surface and distal stem are plasma sprayed with a 200 μm-thick layer of hydroxyapatite of high crystallinity. The stem has a large, straight proximal rectangular portion designed to achieve primary stability via a metaphyseal fit, and has collared and collarless options. The Furlong Evolution^®^ femoral short stem design is based on the Furlong HAC stem retaining the full HAC coating and rectangular proximal body to enable osseointegration and maintain primary stability. The main visual difference is that the stem is notably shorter in length and has a reduced proximal lateral shoulder compared to its predecessor. This potentially has the advantage of allowing it to be more easily introduced with less soft tissue disruption to the patient. Importantly this change in length seems not to have any effect on stress patterns throughout the implant^19^. However, all of these potential characteristics are yet to be confirmed clinically.

## Methods

**Figure 1:**
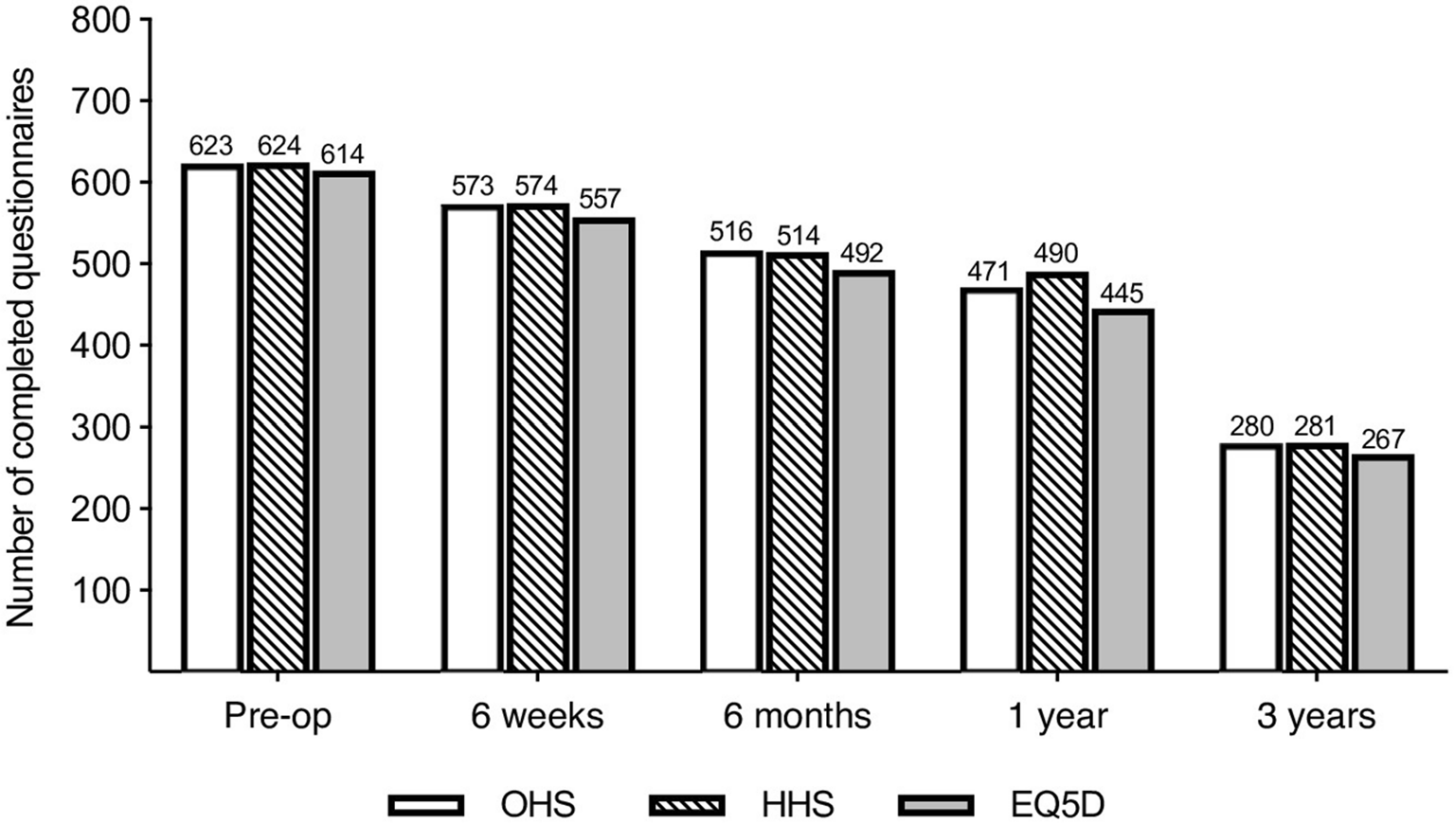
Number of completed questionnaires at each time interval.

Our study cohort consists of a consecutive series of 668 patients who underwent Total Hip Arthroplasty using the Furlong^®^ Evolution HAC femoral stem component (JRI Orthopaedics Ltd, London, UK) between 2012 and 2020. Of the 668 prospective patients recruited 137 withdrew from the study at various time points. The number of patients with completed questionnaires at each time period are demonstrated in Fig. 1.

The surgeries were performed in 12 centers in the United Kingdom by 11 experienced consultant hip surgeons. Patients aged over 18 years who were eligible for total hip arthroplasty were included in this study, with no exclusions related to bone quality or proximal femur morphology (Table 1). 311 patients were male, 357 were female and 9 patients had bilateral hip replacements.

**Table 1:** Inclusion and exclusion criteria for Total Hip Replacement with the Furlong Evolution implant.

Inclusion criteria	Exclusion criteria
Patients consenting to total hip replacement	Patients deemed unsuitable for Total Hip Replacement
Patients deemed suitable for total hip replacement	Patients who refuse to have surgery opting for other modes of treatment
Patients passing pre-assessment deeming them medically fit enough for total hip replacement	Patients who are unable to consent due to a lack of capacity
	Patients unable to understand English
	Any patient under the age of 18 years
	Patients who are pregnant or breast feeding
	Patients deemed unsuitable for Total Hip Replacement

All patients provided their informed consent and attended a pre-operative clinic to ensure they meet the pre-determined inclusion and exclusion criteria. Patient positioning and approach was performed according to surgeon preference and normal practices and broaching and implantation was performed in accordance with the published operative technique. Bearing choice and the use of collared or collarless implants was based on surgeon preference. Intravenous antibiotic prophylaxis and post-operative thromboprophylaxis were administered as per local hospital protocol. All patients had access to physiotherapy and occupational therapy services as per local standard practice.

Patients were reviewed at 6 weeks, 6 months, 1 year, 3 years and 5 years. Clinical function was measured using the Harris Hip Score, completed by a trained clinician. Patient-reported functional outcome was measured using the Oxford Hip Score, and health-related quality of life was measured using the EuroQol-5D visual analogue score^20^. We recorded all adverse events and revision surgeries and the mode of failure within the 5 year period, including patients who withdrew from the study.

The study included a sufficient sample size in accordance with the reporting of outcomes for the Orthopaedic Data Evaluation Panel (ODEP)^21^. All descriptive statistics were expressed as medians and ranges as data from 6 months onwards were found to be non-parametric. A Friedman test was used to determine if there was a significant improvement in each of the patient reported outcome measures at 1, 3 and 5 years post-operatively. A post-hoc Wilcoxon signed-rank test with Bonferroni adjustment was performed for any significant results. SPSS version 14.0 (SPSS Inc, Chicago, IL)^22^ was used for all statistical analyses and R used to produce graphical data^23^. Statistical significance was set at P values of < 0.05.

Ethical approval was given on 15/08/2012 by NRES Committee London—Westminster (Ethics Reference Number: REF 12/LO/0768). All methods were carried out in accordance with relevant guidelines and regulations of the Declaration of Helsinki.

## Results

The mean age in the study group was 62 (range 23–92). 53% of the patients were female and 47% male. The Furlong Evolution Femoral component has a distal size ranging from size 6 mm to 17 mm, all having a stem length of 100 mm. Size 11 stems were implanted most frequently, and no surgeons used sizes greater than 13.

**Figure 2:**
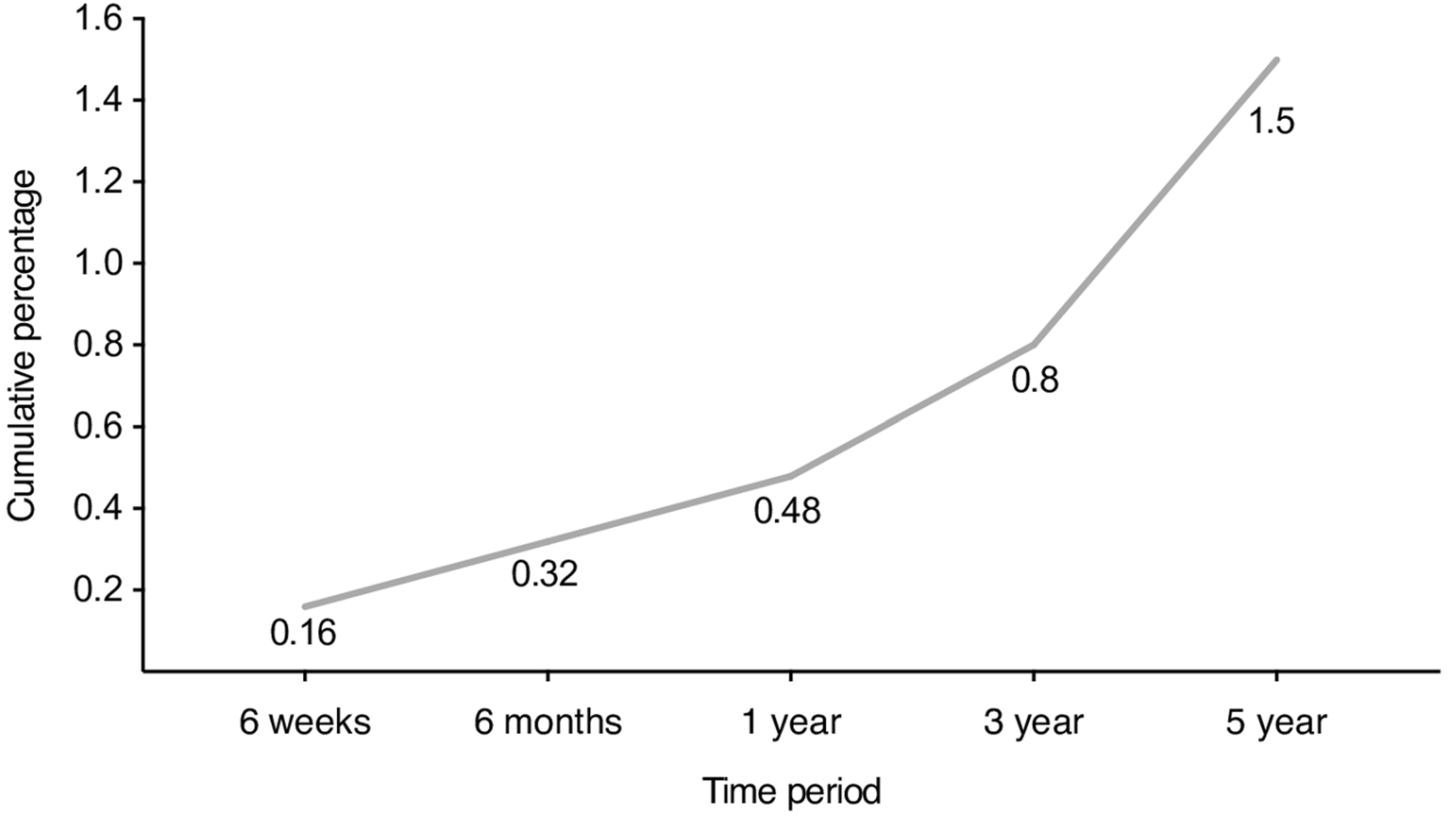
Cumulative revision rates.

Of the 668 patients enrolled 12 patients required revisions; nine of the stem, one of the cup and two patients underwent liner exchange (Table 2) amounting to a cumulative revision rate of 1.8% at 3 years (Fig. 2).

**Table 2:** Summary of patients who underwent a revision.

Revised component	Type	Cause
Stem	Collared	Intraoperative fracture
Stem	Collared	Leg length discrepancy and post-operative hip pain
Stem	Collarless	Peri-prosthetic fracture following fall
Stem	Collared	Post-operative fall
Stem	Collarless	Post-operative fall
Cup	Ceramic	Iliopsoas impingement
Cup	Ceramic	Acetabular fracture
Stem	Unknown	Shaft penetration
Stem	Collarless	Post-operative pain due to small femoral component
Stem	Collarless	Post-operative pain and femoral subsidence
Head and liner	N/A	Low grade infection and subsidence of the femoral component
Head and liner	N/A	Low grade infection and groin pain

Other complications not leading to revision and their incidence are shown in Table 3. Of the patients who did not require revisions seven patients had post-operative hip dislocations, nine patients had wound infections with two patients requiring a wound washout. One patient had a post-operative symptomatic deep vein thrombosis and two patients developed a pulmonary embolism.

**Table 3:** Number of patients experiencing adverse events within 5 years.

Adverse event	Number of patients	Percentage of hip replacements (%)	Number of hip replacements requiring revision
Periprosthetic fracture	14	2.0	6
Subsidence	4	0.6	1
Iliopsoas impingement	1	0.1	1
Dislocation	7	1.0	0
Wound infection	9	1.3	0
Deep infection	2	0.3	2
Thromboembolism	3	0.4	0
Post-operative pain	2	0.3	2
Total	42	6.2	12

**Figure 3:**
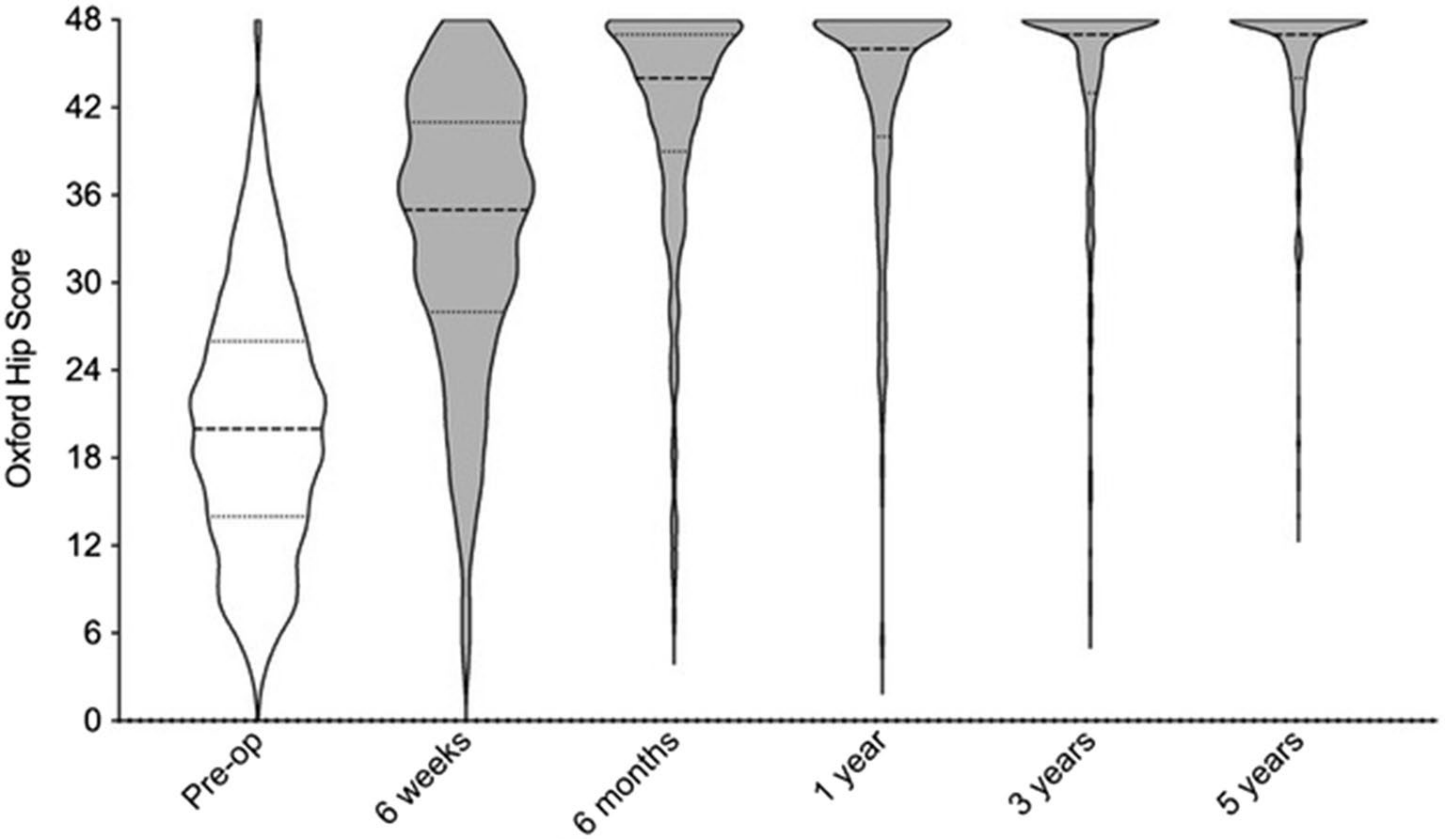
Violin plot of Oxford Hip Scores at each time interval.

The median oxford hip score demonstrated a significant increase from pre-operative levels at 1 year (21 to 46; p < 0.001), 3 years (21 to 47; p < 0.001) and 5 years (21 to 47; p < 0.001) (Fig. 3).

**Figure 4:**
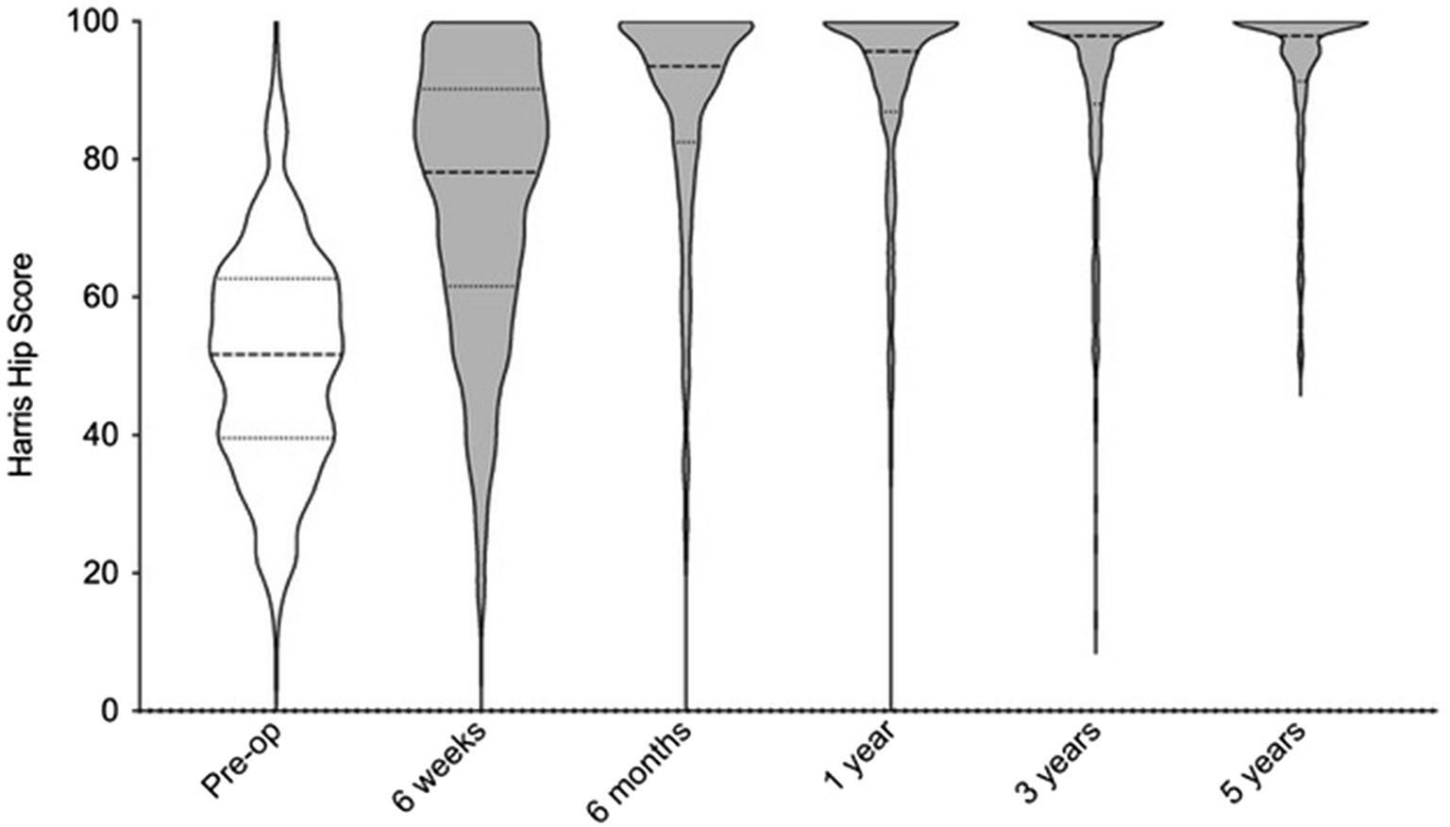
Violin plot of Harris Hip Scores at each time interval.

**Figure 5:**
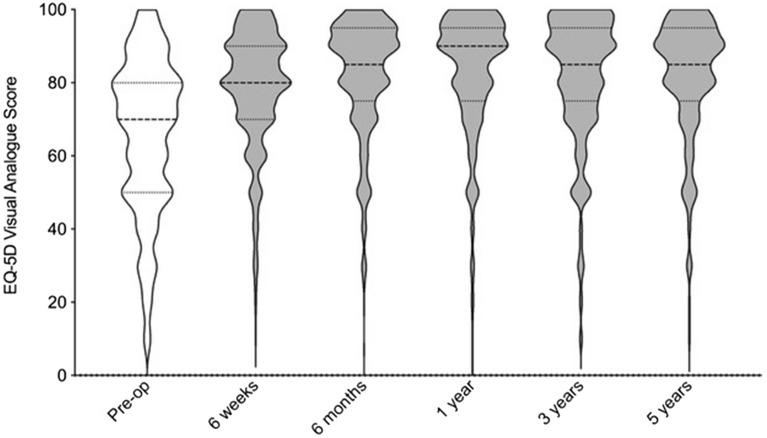
Violin plot of EQ5D-Visual Analogue Scores at each time interval.

The Harris Hip Score improved at 1 year (52 to 98; p < 0.001), 3 years (52 to 98; p < 0.001) and 5 years (52 to 98) (Fig. 4) and EQ5D at 1 year (70 to 90; p < 0.001), 3 years (70 to 85; p < 0.001) and 5 years (70 to 85; p < 0.001) (Fig. 5).

## Discussion

It is well stablished that primary stability in cementless implantation is the major factor to determine bone growth and success of cementless implants^24^ Micromotion at the implant bone interface can affect this stability which, aside from implant positioning and size, can be affected by implant design and patient anatomy^25,26^. Computational modelling has demonstrated high primary stability and tolerance of the Furlong Evolution short stem prosthesis despite variability in patient anatomy^19^. The aim of this study was to clinically assess the safety profile and reported outcome measures of the hydroxyapatite coated Furlong Evolution short stem prosthesis. To our knowledge, this is the largest multicentre consecutive series evaluating reported outcome measures and adverse events using a hydroxyapatite coated short stem femoral component at 5-year follow up. Overall, we found a significant improvement in median EQ5D-VAS, Oxford Hip Scores and Harris Hip Scores and an acceptable safety profile of the short stem Furlong Evolution femoral component at 1 year, 3 years and 5 years in patients who underwent THA.

At 5-year follow up the mean OHS improved from 31 to 45, which is at least as good as post-operative outcomes of uncemented conventional and short stem femoral stems^4^. Stafford et al. retrospectively analysed 250 ceramic-on-ceramic hip replacements demonstrating a mean Oxford Hip Score of 41 at mean 59-month follow up^27^, whilst Hossain et al. reported an increase from 14 to 42 at an average of 31 months follow up in 33 patients using the Taperloc Microplasty (Biomet, Warsaw, Indiana) femoral component^9^. Similarly, a previous study assessing 65 THA patients using the Proxima Hip short stem component (Depuy, Leeds, United Kingdom) demonstrated a mean OHS of 43 at 1.7 years follow up^28^. Our results are also comparable to the National Joint Registry which reports median post-operative OHS of 41 at 18 months post-operatively for uncemented total hip replacement.

Improvements in Harris Hop Scores are also comparable to previous studies. A 2016 meta-analysis compared post-operative HHS between conventional and short stem femoral components^11^. Of the six studies including 552 patients mean HHS ranged from 86 to 96 at between 85 and 95 at follow ups of more than a year which is comparable to our results (median 96 at 1 year and 98 at 5 years). Overall there was no significant difference in HHS between short stems and conventional stems.

Hydroxyapatite coated titanium stems have demonstrated good long-term results in primary hip replacements in both young as well as older age groups^15–17,29–33^. The Furlong^®^ HA C conventional implants have also demonstrated good results with revision rates of less than 10% at 22.5 years^34^. As per the National Institute of Clinical Excellence (NICE) guidelines the best prostheses demonstrate a revision rate of 10% or less at 10 years. This should be regarded as the current benchmark in the selection of prostheses for primary THA. It is also considered reasonable to recommend a prostheses with a maximum of 3% revision rate at 3 years, which would indicate a performance that would then be subjected to annual review to ensure that the revision rate remains consistent with the 10 year benchmark^35^. The Orthopaedic Data Evaluation Panel (ODEP) was established to evaluate data on the outcomes of prostheses provided by manufactures and to inform the NHS which products are compliant with the benchmarks set by NICE. Overall, the stem revision rates are ODEP compliant with rates of 0.7% at 1 year, 1.5% at 3 years and 1.8% at 5 years. Six patients required revisions due to peri-prosthetic fracture of which two were sustained intra-operatively. Only one patient required revision due to subsidence of the femoral stem and two patients had deep infections requiring change of the liner and head. These results are lower than average revision rates recorded in the NJR at 1 year (0.96%), 3 years (1.75%) and 5 years (2.54%) for uncemented implants^4^.

Our study is limited by the relatively short follow up. Figure 2 demonstrates the revision rate is increasing over time although this is difficult to conclude due to the short follow up. Longer term follow up is required and will be performed at 7 and 10 years to further assess the safety profile of this stem. We also did not use radiograph results as a primary outcome for this study, however as bone remodelling and tissue modifications are related to implant design long-term radiograph assessment of femoral stems is often difficult and heterogenous. Comparatively PROMs and revision rates are common clinically relevant metrics of performance. 29% of patients demonstrated a ceiling effect for Oxford Hip Score measurements at 1 year, as such functional outcomes in some of these patients may be better than reported.

As a multi-centre study there may variations in certain operative practices between hospitals. To limit this all patients underwent a standard pre-operative assessments, post-operative care and clinic follow up as per our research protocol. There was also no limit on patient selection. All surgeons followed the published operative technique. We also did not compare short stem results to conventional stems in this study, although the aim of this study is not to demonstrate superiority to the standard length stem but rather determine the safety profile of the short stem. The functional outcomes and survivorship data of the Furlong HAC conventional stem is well published, however, and our results are comparable, both in terms of function and survivorship of the Furlong HAC stem as reflected in the National Joint Registry^35^. Wiik et al. also evaluated the lower limb gait loading differences in patients with a short stem evolution stem and a Furlong conventional stem on the contralateral side with no difference in loading patterns^26^.

Overall, we demonstrate excellent patient reported outcome measures at 5-year follow up using the uncemented Furlong short stem component as well as an acceptable safety profile at 5 years post-operatively. Whilst these results demonstrate short-term safety and effectiveness, longer term follow up of short stem components are required to assess the impact of stem length on survivorship and function in these patients.

## Data Availability

The datasets used and/or analysed during the current study are available from the corresponding author on reasonable request.
